# Vaccination contre la dengue Avis de la Société Francophone de Médecine Tropicale et Santé Internationale concernant la France métropolitaine et les territoires ultramarins

**DOI:** 10.48327/mtsi.v4i4.2024.603

**Published:** 2024-11-17

**Authors:** Yves BUISSON, Éric PICHARD

**Affiliations:** 1 Membre de l’Académie nationale de médecine, SFMTSI Société francophone de médecine tropicale et santé internationale (ancienne SPE), Hôpital Pitié-Salpêtrière, Pavillon Laveran, 47-83 Boulevard de l’Hôpital, 75651 Paris cedex 13, France; 2 Président de la SFMTSI, SFMTSI Société francophone de médecine tropicale et santé internationale (ancienne SPE), Hôpital Pitié-Salpêtrière, Pavillon Laveran, 47-83 Boulevard de l’Hôpital, 75651 Paris cedex 13, France

**Keywords:** Dengue, Expansion mondiale, *Aedes albopictus*, Vaccins (Dengvaxia^®^ et Qdenga^®^), Adultes à risque, Dengue, Global spread, *Aedes albopictus*, Vaccines (Dengvaxia^®^ and Qdenga^®^), Adults at risk

## Abstract

La dengue est en forte expansion mondiale, touchant près de la moitié de la population mondiale. Ses causes incluent l'urbanisation, la mobilité humaine, le changement climatique et la propagation des moustiques vecteurs comme *Aedes albopictus.* En 2023 et 2024, on a observé une hausse marquée des cas et des décès dans le monde. En France métropolitaine, l'accroissement des cas importés a généré des transmissions locales. La dengue est asymptomatique dans plus de 50 % des cas, mais elle peut évoluer vers des formes graves dans 1 à 5 % des cas symptomatiques, avec des complications potentiellement fatales. Il existe quatre sérotypes de virus, et une réinfection par un autre sérotype augmente le risque de forme sévère.

Actuellement, deux vaccins contre la dengue sont disponibles : le Dengvaxia^®^ et le Qdenga^®^ (TAK-003). Le Dengvaxia^®^ est réservé aux personnes déjà infectées par la dengue, mais sa production a cessé en 2024 en raison de la faible demande. Le Qdenga^®^ est recommandé pour les enfants de 6 à 16 ans dans les zones de transmission élevée. La SFMTSI propose une extension de la vaccination aux adultes à risque dans les territoires ultramarins endémiques et aux voyageurs. Une campagne de communication est suggérée pour informer le public des bénéfices de la vaccination tout en anticipant les risques de désinformation antivaccinale.

## Abstract (en)

Dengue fever is spreading rapidly around the world, affecting nearly half the world's population. Causes include urbanization, human mobility, climate change and the spread of mosquito vectors such as *Aedes albopictus.* In 2023 and 2024, there was a marked increase in cases and deaths worldwide. In mainland France, the increase in imported cases has generated local transmissions.

Dengue fever is asymptomatic in over 50% of cases, but can progress to severe forms with potentially fatal complications in 1-5% of symptomatic cases. There are four serotypes of the virus, and re-infection with another serotype increases the risk of severe disease.

Two dengue vaccines are currently available: Dengvaxia^®^ and Qdenga^®^ (TAK-003). Dengvaxia^®^ is reserved for people already infected with dengue fever, but production was discontinued in 2024 due to low demand. Qdenga^®^ is recommended for children aged 6-16 years in high transmission areas. The Société francophone de Médecine tropicale et Santé internationale (SFMTSI) proposes extending vaccination to at-risk adults in endemic overseas territories and to travelers. A communication campaign is proposed to inform the public about the benefits of vaccination while anticipating the risks of anti-vaccination misinformation.

## Données épidémiologiques

La dengue est en expansion dans le monde. Plusieurs déterminants favorisent cette tendance : l'urbanisation avec forte densité de population, la mobilité humaine, le changement climatique et l'extension de l'aire de répartition des vecteurs, notamment celle *d’Aedes albopictus* (« moustique tigre »). Selon l’OMS, près de la moitié de la population mondiale est aujourd'hui exposée à l'infection. Une modélisation récente évalue à 390 millions le nombre d>infections dans le monde au cours d'une année, dont 96 millions de malades, un demi-million d>hospitalisations et 20 000 morts dont une très forte proportion d>enfants. La dengue est endémique dans 125 pays situés dans les régions tropicales et subtropicales du monde, les Amériques, l’Asie du Sud-Est et le Pacifique occidental étant les régions les plus gravement touchées.

L'année 2023 correspond à une augmentation spectaculaire des taux d'incidence et des décès notifiés. Cette recrudescence s'observe aussi dans les pays non endémiques en raison d'une augmentation singulière du nombre de cas importés. Ainsi, depuis 2023, la France métropolitaine connaît une situation sans précédent, liée en majorité à des cas importés des Antilles où une épidémie de dengue sévit depuis le mois de juillet 2023. Cette situation s'est aggravée en 2024 avec 2 271 cas notifiés par déclaration obligatoire du 1^er^ janvier au 30 avril. S'y ajoute le risque de transmission autochtone, la présence *d’Aedes albopictus* étant confirmée dans 74 départements de métropole. Une cinquantaine de cas autochtones y ont été notifiés en 2023 et 57 cas pendant le seul été 2024 (Santé publique France).

La dengue n’épargne pas les voyageurs. Le réseau mondial Geo-Sentinel a relevé 5 958 cas de 2007 à 2022 chez des voyageurs des pays du Nord revenant de zones endémo-épidémiques. Bien qu'il n'y ait eu que 0,5 % de cas sévères, le taux d'hospitalisation était de 26,7 %. Outre le risque individuel de dengue sévère lié aux facteurs favorisants, le risque collectif est l'apparition d'une transmission de la dengue dans les zones non endémiques comme la France métropolitaine où *A. albopictus* s'est implanté.

La dengue est asymptomatique dans plus de 50 % des cas, mais des formes sévères peuvent s'observer dans 1 à 5 % des cas symptomatiques. Elles résultent d'une augmentation de la perméabilité vasculaire qui entraîne une fuite plasmatique pouvant évoluer vers l’état de choc et mettre en jeu le pronostic vital. Il existe aussi des formes graves d'expression hémorragique, neurologique ou hépatique.

Le virus de la dengue comporte quatre sérotypes (DENV-1, DENV-2, DENV-3 et DENV-4). L'infection par l'un de ces sérotypes (dengue primaire) n'immunise pas contre les trois autres. Au contraire, le risque de développer une forme grave semble plus important lorsqu'un individu est réinfecté par un autre sérotype (dengue secondaire). Les autres facteurs de risque de dengue sévère sont la grossesse (surtout au troisième trimestre), les âges extrêmes (moins de 2 ans ou plus de 65 ans), les pathologies chroniques (diabète, obésité, insuffisance cardiaque, asthme, hépatopathies chroniques, syndromes drépanocytaires majeurs) et la prise d'anticoagulants.

## Vaccins contre la dengue

Jusqu’à présent, deux vaccins contre la dengue ont été préqualifiés par l’OMS, le Dengvaxia^®^ (Sanofi) et le TAK-003 ou Qdenga^®^ (Takeda). Ce sont des vaccins vivants, atténués, chimériques, recombinants et quadrivalents, dirigés contre les quatre sérotypes.

Le CYD-TDV ou Dengvaxia^®^ est un vaccin chimérique préparé à partir du vaccin antiamaril 17D. Il a reçu ses premières autorisations de mise sur le marché en 2015. En raison d'un risque accru de dengue sévère chez les sujets n'ayant pas d'antécédent de dengue, Dengvaxia^®^ était indiqué uniquement pour les personnes âgées de 9 à 45 ans, ayant déjà été infectées par un virus de la dengue et vivant en zone endémique. Du fait de ces limitations et malgré son efficacité, la production de ce vaccin a été arrêtée le 31 mars 2024, la demande pour ce vaccin étant trop faible au niveau mondial.

Le TAK-003 ou Qdenga^®^ (Takeda) est aussi un vaccin chimérique, préparé à partie d'une souche DENV-2 atténuée. Il doit être administré selon un schéma en deux doses à trois mois d'intervalle. Une étude de phase 3, randomisée contre placebo, réalisée chez 20 000 enfants et adolescents dans huit pays endémiques d’Amérique et d’Asie conclut à une efficacité cumulative de 61,2 % contre la dengue confirmée virologiquement (DCV) et de 84,1 % contre la DCV hospitalisée. Cette efficacité est plus faible chez les participants séronégatifs avant vaccination : 53,5 % et 79,3 % respectivement. Une stratification selon l’âge conclut à l'efficacité du TAK-003 dans la prévention de la dengue en zones d'endémie dans toutes les tranches d’âge de 4 à 16 ans, avec des différences suivant le sérotype. On ne dispose pas d’études d'efficacité vaccinale chez l'adulte, mais seulement d'une étude d'immunogénicité jusqu’à l’âge de 45 ans montrant une persistance des anticorps neutralisants à trois ans, avec des titres plus élevés chez les séropositifs à l'inclusion que chez les séronégatifs. En termes de sécurité, les taux d’évènements indésirables graves ont été identiques dans les groupes vaccin et placebo, indépendamment du statut sérologique à l'inclusion. Chez les enfants âgés de moins de quatre ans, la sécurité et l'efficacité de Qdenga^®^ n'ont pas été établies.

Deux autres vaccins tétravalents vivants atténués contre la dengue sont en cours de développement :

- le vaccin brésilien Butantan-Dengue (Butantan-DV) est administré en dose unique. Un essai de phase 3 contre placebo stratifié selon l’âge (2-6 ans, 7-17 ans et 18-59 ans) réalisé au Brésil a montré une efficacité clinique globale de 80,1 % entre 2 et 6 ans, de 77,8 % entre 7 et 17 ans et de 90,0 % entre 18 et 59 ans vis-à-vis des 2 sérotypes circulants (DENV-1 et DENV-2) quel que soit le statut sérologique initial.
- le vaccin TV005 (National Institute of Allergy and Infectious Diseases) est également utilisé en dose unique. Dans un essai de phase 2 réalisé au Bangladesh avec un suivi de trois ans dans différentes classes d’âge jusqu’à 50 ans, le TV005 s'est avéré bien toléré et immunogène pour les quatre sérotypes chez les jeunes enfants et les adultes, y compris chez les personnes n'ayant jamais été exposées à la dengue.

## Recommandations actuelles

La Commission européenne a accordé une autorisation de mise sur le marché (AMM) du vaccin TAK-003 le 8 décembre 2022 pour les personnes âgées de quatre ans et plus, indépendamment d'une exposition antérieure à l'infection.

L’OMS a préqualifié le vaccin TAK-003 le 10 mai 2024; elle recommande de l'utiliser chez les enfants âgés de 6 à 16 ans dans les zones où l'intensité de la transmission de la dengue est élevée. Avant de formuler un avis définitif, la Haute autorité de santé (HAS) française a recommandé le 4 juillet 2024 la vaccination contre la dengue par le vaccin Qdenga^®^ pour les enfants et adolescents qui réunissent les conditions suivantes :

- être âgé de 6 à 16 ans;
- résider dans les territoires français d’Amérique (Antilles et Guyane);
- avoir la preuve documentée d'une dengue antérieure (infection biologiquement confirmée en laboratoire par RT-PCR ou détection de l'antigène NS1) ou mention d'une infection cliniquement diagnostiquée en contexte épidémique dans le carnet de santé.

Ces recommandations excluent les adultes, les voyageurs ainsi que les personnes résidant à la Réunion, à Mayotte et dans les territoires de l’Océan Pacifique (Nouvelle-Calédonie, Wallis et Futuna, Polynésie française).

## Avis de la Société Francophone de Médecine Tropicale et Santé Internationale (SFMTSI)

La recrudescence mondiale de la dengue et son extension rapide vers des zones potentiellement endémiques imposent la mise en œuvre d'un renforcement des mesures préventives et l'adoption d'une stratégie vaccinale. Celle-ci est rendue possible par la mise à disposition de nouveaux vaccins, le vaccin TAK-003 (Qdenga^®^) étant actuellement le seul vaccin contre la dengue autorisé et disponible.

Compte-tenu des données d'efficacité et d'innocuité issues des essais cliniques, la SFMTSI propose l’élargissement de la vaccination par deux doses de Qdenga^®^ :

- aux enfants à partir de l’âge de quatre ans, résidant dans les territoires ultramarins endémiques (Antilles, Guyane, Réunion, Mayotte, Nouvelle Calédonie, Polynésie française, Wallis-et-Futuna);
- aux adultes résidant dans ces territoires et présentant des facteurs de risque de dengue sévère (antécédents de dengue, âge égal ou supérieur à 65 ans, obésité, diabète, insuffisance cardiaque, asthme, hépatopathies, hémoglobinoses et thalassémies majeures, troubles de la coagulation et traitements anticoagulants), à l'exclusion des femmes enceintes ou allaitantes et des malades immunodéprimés;
- aux voyageurs âgés de quatre ans et plus, consultant dans les Centres de vaccination internationale (CVI) français, qui projettent de se rendre dans une zone où la dengue est endémique ou épidémique.

La SFMTSI souligne la nécessité de promouvoir une campagne de communication, avant et pendant la mise en œuvre de ces recommandations vaccinales, afin d'informer le public des avantages et bénéfices dans le contexte épidémiologique actuel, tout en précisant les limites d'efficacité et les effets secondaires potentiels. Il est essentiel d’éviter la propagation de rumeurs antivaccinales qui pourraient freiner l'adhésion à cette nouvelle mesure de prévention des personnes exposées à la dengue, ainsi qu'aux autres programmes de vaccination..

