# Supplementary material for: Dengue fever vaccination Recommendations from the Société Francophone de Médecine Tropicale et de Santé Internationale for Metropolitan France and Overseas Territories
Source: Med Trop Sante Int. 2024 Nov 17;4(4):mtsi.v4i4.2024.603. [Article in French] doi: 10.48327/mtsi.v4i4.2024.603 (PMC11892382; doi:10.48327/mtsi.v4i4.2024.603)
Supplement: Related texts

# Research article: Dengue fever vaccination Recommendations from the Société Francophone de Médecine Tropicale et de Santé Internationale for Metropolitan France and Overseas Territories

## Epidemiological data

Dengue fever is increasing worldwide. A number of factors are driving this trend: dense urbanization, human mobility, climate change, and the expansion of the natural range of vectors, particularly of *Aedes albopictus* (“tiger mosquito”). According to the WHO, almost half of the world's population is now exposed to infection. A recent model estimates the number of infections worldwide at 390 million per year, including 96 million cases, half a million hospitalizations, and 20,000 deaths. A very high proportion of hospitalized ones are children. Dengue fever is endemic in 125 countries in the tropical and subtropical regions of the world. The Americas, Southeast Asia and the Western Pacific are the most affected.

The year 2023 represents a spectacular increase in reported incidence rates and deaths. This increase is also observed in non-endemic countries due to an amazing increase in the number of imported cases. Since 2023, metropolitan France is experiencing an unprecedented situation, mainly due to cases imported from the West Indies, where a dengue epidemic has been raging since July 2023. The situation worsened in 2024, with 2,271 notifiable cases between January 1 and April 30. There is also the risk of autochthonous transmission, with the presence of *Aedes albopictus* confirmed in 74 French metropolitan departments. About 50 indigenous cases were reported in 2023 and 57 cases in the summer of 2024 alone (Santé publique France).

Dengue does not spare travelers. The global Geo-Sentinel network recorded 5,958 cases between 2007 and 2022 among travelers from northern countries returning from endemic areas. Although only 0.5% of cases were severe, the hospitalization rate was 26.7%. In addition to the individual risk of severe dengue fever, the collective risk is the emergence of dengue transmission in non-endemic areas, such as metropolitan France, where *A. albopictus* has become established. Dengue is asymptomatic in over 50% of cases, but severe forms can occur in 1% to 5% of symptomatic cases. These result from an increase in vascular permeability leading to plasma leakage, which can progress to shock and be life-threatening. There are also severe forms with hemorrhagic, neurologic or hepatic manifestations.

Dengue virus consists of four serotypes (DENV-1, DENV-2, DENV-3 and DENV-4). Infection with one of these serotypes (primary dengue) does not confer immunity to the other three. On the contrary, the risk of developing severe dengue seems to be higher if a person is re-infected with another serotype (secondary dengue). Other risk factors for severe dengue include pregnancy (especially in the third trimester), extreme age (under 2 or over 65 years of age), chronic pathologies (diabetes, obesity, heart failure, asthma, chronic liver disease, severe sickle cell disease) and anticoagulants.

## Dengue vaccines

To date, two dengue vaccines have been prequalified by the WHO: Dengvaxia^®^ (Sanofi) and TAK-003 or Qdenga^®^ (Takeda). These are live, attenuated, chimeric, recombinant and quadrivalent vaccines against the four serotypes. CYD-TDV or Dengvaxia^®^ is a chimeric vaccine based on the 17D yellow fever vaccine. It received its first approvals in 2015. Due to an increased risk of severe dengue fever in individuals without a history of dengue fever, Dengvaxia^®^ was indicated only for people aged 9 to 45 years who had been previously infected with dengue virus and living in endemic areas. As a result of these limitations and despite its efficacy, production of this vaccine was discontinued on March 31, 2024, due to insufficient global demand.

TAK-003 or Qdenga^®^ (Takeda) is also a chimeric vaccine made from an attenuated strain of DENV2. It is administered in two doses, three months apart. A Phase 3 randomized, placebo-controlled study in 20,000 children and adolescents in eight endemic countries in the Americas and Asia showed a cumulative efficacy of 61.2% against virologically confirmed dengue fever (VCD) and 84.1% against hospitalized VCD. This efficacy was lower in seronegative participants prior to vaccination: 53.5% and 79.3%, respectively. Age stratification showed that TAK-003 was effective in preventing dengue fever in endemic areas in all age groups from 4 to 16 years, with differences by serotype. There are no vaccine efficacy studies in adults, only an immunogenicity study up to 45 years of age. It showed persistence of neutralizing antibodies at three years, with higher titers in seropositive at enrollment than in seronegative subjects. In terms of safety, the rates of serious adverse events were identical in the vaccine and placebo groups, regardless of serologic status at enrollment. The safety and efficacy of Qdenga^®^ have not been established in children under four years of age.

Two other tetravalent, live-attenuated dengue vaccines are currently in development:

- the Brazilian single-dose vaccine Butantan-Dengue (Butantan-DV). A placebo-controlled phase 3 trial conducted in Brazil, stratified by age (2-6 years, 7-17 years and 18-59 years), showed overall clinical efficacy of 80.1% between 2 and 6 years, 77.8% between 7 and 17 years and 90.0% between 18 and 59 years against the 2 circulating serotypes (DENV-1 and DENV-2), regardless of initial serologic status.
- the TV005 vaccine (National Institute of Allergy and Infectious Diseases) is also used as a single dose. In a Phase 2 study conducted in Bangladesh with a three-year follow-up in various age groups up to 50 years, TV005 was shown to be well tolerated and immunogenic against all four serotypes in young children and adults, including those with no previous exposure to dengue.

## Current recommendations

The European Commission granted marketing authorization (MA) for TAK-003 on December 8, 2022 for people four years of age and older, regardless of prior exposure to infection.

On May 10, 2024, the WHO prequalified TAK-003 vaccine and recommended its use in children aged 6 to 16 years in areas with intense dengue transmission.

On July 4, 2024, the French Haute Autorité de Santé (HAS), before issuing its final opinion, recommended dengue vaccination with the Qdenga^®^ vaccine for children and adolescents who:

- are between 6 and 16 years of age;
- reside in the French territories of the Americas (French West Indies and French Guiana);
- have documented evidence of previous dengue fever (biologically confirmed infection by laboratory RT-PCR or NS1 antigen detection) or mention in their medical records of clinically diagnosed infection in the context of an epidemic.

These recommendations exclude adults, travelers, and residents of Reunion Island, Mayotte, and the Pacific territories (New Caledonia, Wallis and Futuna, French Polynesia).

## Statement of the Société Francophone de Médecine Tropicale et Santé Internationale (SFMTSI)

The global resurgence of dengue fever and its rapid spread to potentially endemic areas necessitates the strengthening of preventive measures and the adoption of a vaccine strategy. TAK-003 (Qdenga^®^) is currently the only licensed and available dengue vaccine.

Based on the efficacy and safety data from clinical trials, the SFMTSI proposes to expand vaccination with two doses of Qdenga^®^ to:

- children aged four years and older living in endemic overseas territories (French West Indies and Guiana, Reunion Island, Mayotte, New Caledonia, French Polynesia, Wallis and Futuna);
- adults residing in these territories with risk factors for severe dengue fever (history of dengue fever, age 65 years or older, obesity, diabetes, heart failure, asthma, liver disease, hemoglobinopathies and major thalassemias, coagulation disorders and anticoagulant treatment), excluding pregnant or breastfeeding women and immunocompromised patients;
- travelers aged four years and older who consult the French International Vaccination Centres (CVI) and who plan to visit an area where dengue is endemic or epidemic.

The SFMTSI stresses the need to promote a communication campaign, before and during the implementation of these vaccination recommendations, to inform the public of the advantages and benefits in the current epidemiological context, while specifying the limits of efficacy and potential side effects. It is essential to avoid the spread of anti-vaccination rumors, which could slow down the uptake of this new preventive measure, as well as of other vaccination programs.
